# cROStalk for Life: Uncovering ROS Signaling in Plants and Animal Systems, from Gametogenesis to Early Embryonic Development

**DOI:** 10.3390/genes12040525

**Published:** 2021-04-03

**Authors:** Valentina Lodde, Piero Morandini, Alex Costa, Irene Murgia, Ignacio Ezquer

**Affiliations:** 1 Reproductive and Developmental Biology Laboratory, Department of Health, Animal Science and Food Safety (VESPA), Università degli Studi di Milano, 20133 Milan, Italy; valentina.lodde@unimi.it; 2 Department of Environmental Science and Policy, Università degli Studi di Milano, 20133 Milan, Italy; piero.morandini@unimi.it; 3 Department of Biosciences, Università degli Studi di Milano, 20133 Milan, Italy; alex.costa@unimi.it (A.C.); irene.murgia@unimi.it (I.M.)

**Keywords:** reactive oxygen species, calcium signaling, reproductive development in plants and mammals, gametogenesis, embryo development, signal transduction

## Abstract

This review explores the role of reactive oxygen species (ROS)/Ca^2+^ in communication within reproductive structures in plants and animals. Many concepts have been described during the last years regarding how biosynthesis, generation products, antioxidant systems, and signal transduction involve ROS signaling, as well as its possible link with developmental processes and response to biotic and abiotic stresses. In this review, we first addressed classic key concepts in ROS and Ca^2+^ signaling in plants, both at the subcellular, cellular, and organ level. In the plant science field, during the last decades, new techniques have facilitated the in vivo monitoring of ROS signaling cascades. We will describe these powerful techniques in plants and compare them to those existing in animals. Development of new analytical techniques will facilitate the understanding of ROS signaling and their signal transduction pathways in plants and mammals. Many among those signaling pathways already have been studied in animals; therefore, a specific effort should be made to integrate this knowledge into plant biology. We here discuss examples of how changes in the ROS and Ca^2+^ signaling pathways can affect differentiation processes in plants, focusing specifically on reproductive processes where the ROS and Ca^2+^ signaling pathways influence the gametophyte functioning, sexual reproduction, and embryo formation in plants and animals. The study field regarding the role of ROS and Ca^2+^ in signal transduction is evolving continuously, which is why we reviewed the recent literature and propose here the potential targets affecting ROS in reproductive processes. We discuss the opportunities to integrate comparative developmental studies and experimental approaches into studies on the role of ROS/ Ca^2+^ in both plant and animal developmental biology studies, to further elucidate these crucial signaling pathways.

## 1. Introduction

In response to environmental stimuli, plants are able to produce reactive oxygen species (ROS) to control many and different physiological processes, such as responses to biotic stresses, activation of programmed cell death (PCD), germination, regulation of stomatal opening, sexual reproduction, or flowering time (see the vast literature available [1,2,3,4,5,6,7,8,9]). These are just some examples of the distinct processes controlled by ROS metabolism. Nevertheless, ROS by-products are generated in almost all cellular compartments, ranging from chloroplasts to mitochondria and peroxisomes, but also in the cell walls and plasma membrane, apoplast, cytosol, and glyoxysomes (for a complete overview, see [10]). Being ubiquitous, ROS reactions induce fast and dramatic effects on the growth of multiple plant tissues and organs in order to modify patterns of growth and morphogenesis. In this review, we provide a general view on ROS and Ca^2+^ signal transduction in plants, both at the subcellular, cellular, and organ level. We also provide a trans-kingdom comparative view, discussing recent advances in ROS studies in animal cells, and a comparative perspective on sexual reproduction and embryo development in both lineages. In plant and animal science, during the last decades, specific sensors have been developed that allow the in vivo detection of ROS and Ca^2+^ dynamics. We describe how these powerful techniques can provide novel perspectives in developmental studies. We discuss how the evolution of these techniques and also omics data can facilitate understanding ROS signaling and signal transduction. Comparative studies in plant and animal reproductive processes will give us a fruitful, comparative, and mechanistic view, taking into the account the similarities present between them. We discuss recent advances in both lineages, and propose novel key players affecting ROS and Ca^2+^ signal transduction, which are to be explored in the future.

## 2. ROS and Physiological and Cellular Responses in Plants

### 2.1. Reactive Oxygen Species: Production and Detoxification

Several ROS exist in biological systems, with a different reactivity and in different concentrations. They are reactive molecules and are therefore potentially toxic. In this section, we first consider the main ones that are produced in plant and animal cells, the antioxidant systems dedicated to their detoxification, and their role as signals.

**Figure 1:**
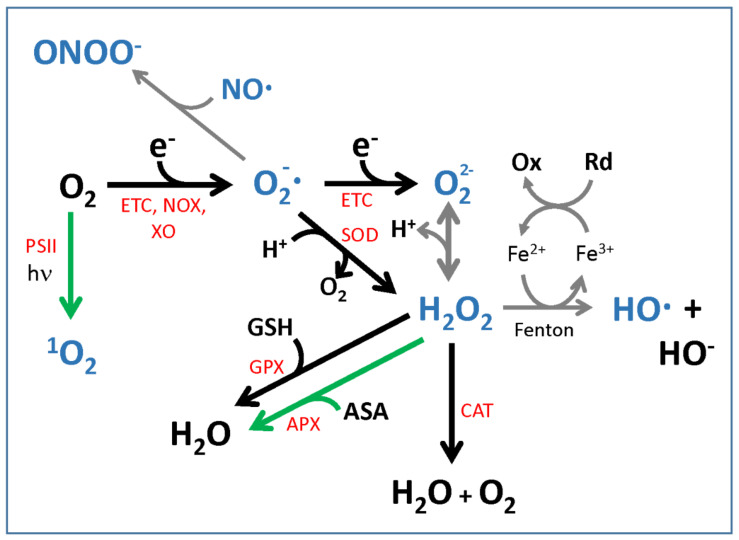
Simplified scheme of cellular (reactive oxygen species/reactive nitrogen species) ROS/RNS metabolism. Reactive species are in blue, and the other reactants in black. Black arrows represent the enzyme-catalyzed reactions, green arrows are essentially plant-specific, grey arrows represent non-enzymatic (spontaneous) reactions, and the enzyme or enzyme complexes are in red. The spontaneous Fenton reaction involves Fe^3+^/Fe^2+^ (or Cu^2+^/Cu^+^, not included) to produce the hydroxyl radical. hν represents the energy of a photon. NOX, NADPH oxidase (which includes plant respiratory burst oxidase homologue (RBOH); XO, xanthine oxidase; Ox and Rd represent any redox couple able to oxidize Fe^2+^; other abbreviations are as in the main text.

ROS are oxygen-derived chemical species (by energy or electron transfer) that are more reactive than oxygen itself and include superoxide anion (O_2_^−^), hydroxyl radical (HO^.^), singlet oxygen (^1^O_2_), hydrogen peroxide (H_2_O_2_), and the related deprotonated anionic forms (HO_2_^−^ and O_2_^2−^) [11,12,13] (Figure 1). Nitric oxide (NO) and peroxynitrite (ONOO^−^), though being reactive oxygen-containing chemical species, are not considered ROS and are normally referred to as reactive nitrogen species (RNS), whose metabolism is, however, intertwined with ROS metabolism (Figure 1); for this reason, they are included in this review, with a primary focus on those species able to act as signaling molecules. The hydroxyl radical OH^.^ reacts at a diffusion-limited rate and its half-life is in the order of ns [12,14]; this ROS species can therefore hardly act as a signaling molecule. Singlet oxygen ^1^O_2_ has also a very limited half-life, i.e., around 1–4 µs [12]; nevertheless, its stress-signaling activity has been well documented [15,16]. The lifetime of superoxide anion is in the µs–ms range, while that of H_2_O_2_ is in the ms–s range, depending on the activities of the scavenging systems [12,17,18]. 

ROS are routine byproducts of cellular metabolism, in physiological conditions. Both superoxide and H_2_O_2_ are indeed produced by the respiratory electron transport chain (ETC), in plant and animal mitochondria; their production rates, however, increase, when ATP consumption slows down or when transport in the respiratory chain is inhibited. Similarly, singlet oxygen and superoxide anions are produced by the photosynthetic ETC in chloroplasts on the PSII side [14,15,19]; H_2_O_2_ is produced in various subcellular compartments in plants [20]. Besides mitochondria and chloroplasts [21,22], ROS also can be produced in various other cellular compartments, such as peroxisomes [23], the plasma membrane, plant cell wall, etc. [10].

One ROS may generate other ROS, either through enzymatic or spontaneous reactions. Examples of this are the production of H_2_O_2_ from superoxide catalyzed by superoxide dismutases (SOD), the reaction of H_2_O_2_ with Fe(II) to produce the hydroxyl radical, or of the superoxide anion with Fe(III) to produce Fe(II), which, in turn, can react with H_2_O_2_ (for a review on ROS chemistry, see [13]). These reactions often lead to the generation of more active, and therefore more damaging, ROS [17]; however, their impact also depends on the presence of free iron (Fe^2+^/Fe^3+^) or other ROS/RNS [24,25]. One ROS may even stimulate signal transduction pathways leading to further ROS generation, for instance through the respiratory-burst NADPH oxidases present in both animal and plant cells [18,26]. Some ROS are able to cross membranes, either by diffusion or by facilitated transport through aquaporins [27]. They can therefore act as signals of the oxidative status to compartments other than the one where they are produced [17,18].

Ozone (O_3_) is also a relevant ROS for both plants and animals, mainly in polluted environments. O_3_ sensing, its effects, reaction products, and plant responses are quite complex [28,29,30,31,32] and go beyond the goal of the present review.

Although ROS are constitutive by-products of essential functions, such as respiration in mitochondria, photosynthesis, and photorespiration in chloroplasts/peroxisomes [33], their production nevertheless increases during exposure to biotic and abiotic stresses, which therefore are referred to as “oxidative stresses” [34]. In particular, the sessile nature of plants forces them to heavily rely on stress resilience strategies rather than stress avoidance [35]. Therefore, plant cells, more than animal cells, must keep steady-state levels of the various ROS under strict control, to avoid any ROS damaging effect on the macromolecules (lipids, DNA, and proteins). This control is achieved through a well-equipped antioxidant system that adjusts, in a coordinated and finely regulated manner, the concentration of various ROS species to avoid ROS noxious effects. For that, two main classes of antioxidant defenses come into play: enzymes and small metabolites (both water and lipid soluble). 

In plant cells, glutathione (the tripeptide γ-glutamyl-cysteinyl-glycine, GSH) [12] and ascorbic acid (ASA), also known as vitamin C (vit. C), are the major metabolites impacting the control of the redox status [12,36,37,38,39,40]. ASA is present in all subcellular compartments of both plants and animals [41,42,43], i.e., the cytosol, nucleus, chloroplasts, mitochondria, peroxisomes, vacuoles, and also in the extracellular fluids of animals and cell wall and apoplast of plants; whereas, GSH accumulates in the cytosol, nucleus, chloroplasts, mitochondria, and peroxisomes ([36] and references therein). The ASA biosynthetic pathway mainly proceeds, in higher plants, through the L-galactono lactone intermediate; this pathway is known as the L-galactose or the Wheeler–Smirnoff pathway [44,45,46,47]. The L-gulono lactone pathway also has been investigated as a potential alternative route of ASA synthesis in plants [48,49]; however, in vivo relevance of this latter pathway could not be confirmed, so far [43,50]. The GSH biosynthetic pathway consists of two enzymes, namely γ-glutamylcysteine synthetase (γ-ECS) and glutathione synthase GSHS [51]. Besides ASA and GSH, other metabolites also can act as antioxidant molecules, such as carotenoids [52], tocopherol, and flavonoids ([36] and references therein). All the enzymes involved in the biosynthesis of these antioxidant metabolites can be considered, though indirectly, as part of the antioxidant enzymatic system. Moreover, various enzymes are directly involved in ROS scavenging, such as those taking part in the ascorbate–glutathione pathway, also known as the “Foyer–Halliwell–Asada’’ pathway [14,39], i.e., superoxide dismutases (SOD) and ascorbate peroxidases (APX) dismutate superoxide into H_2_O_2_ and O_2_ and reduce H_2_O_2_ to H_2_O, respectively; the enzymes monodehydroascorbate reductases (MDAR) and dehydroascorbate reductases (DHAR) replenish, in such a pathway, the pool of reduced ASA (from oxidized ASA, i.e., from monodehydroascorbic acid MDHA and dehydroascorbic acid DHA), whereas glutathione reductases restore the pool of glutathione in its reduced state GSH [14,36,39]. Plants cells are equipped with an array of other antioxidant enzymes, such as peroxiredoxins (PRX) and thioredoxins (TRX); a detailed list is discussed in [36].

ROS are therefore constantly removed by specific enzymes and metabolites. These antioxidant systems reduce the toxic effects of ROS, consisting of the oxidation of sugars, nitrogen bases, lipids, and amino acids; the antioxidant systems therefore prevent extensive damage to DNA, RNA, protein, and membrane function. On the other side, ROS, and similar species, can function as signals of stress and induce specific responses in cells.

### 2.2. ROS Chemistry and Implications for Signaling

The difference between production and scavenging determines the overall result: damage, protection, or signaling [17,18]. The ability of a given ROS to act as a signal between or within compartments depends on its physico-chemical characteristics (for general issues on the chemistry of ROS and the implications for signaling, the reader is referred to recent reviews [18,53,54,55]). For instance, O_3_ and singlet oxygen are not produced in plant mitochondria, while in animals, singlet oxygen is produced only at specific sites, such as in the retina, or during the respiratory burst of neutrophils and eosinophils [56]; so, these ROS cannot be signals of general relevance. On the other hand, superoxide, HO^.^, and H_2_O_2_ are almost ubiquitously produced, but the short half-life of the hydroxyl radical and the lack of known scavengers make it an unlikely signal among compartments. Superoxide, a poorly oxidizing agent in general, is produced in almost every compartment, but is either quickly removed by SOD or it reacts with NO, when available, to form peroxynitrite, which is extremely reactive and able to modify several types of molecules (transition metal centers in proteins, several amino acids (Cys, Tyr, Met, Trp, and His), lipids, and nucleic acids) [24]. SOD-mediated removal of superoxide prevents its reaction with NO [57]. H_2_O_2_ has characteristics that make it ideal for signaling: first, it has a relatively long half-life (1 ms or longer [58]) because it is less reactive than most of the other ROS. H_2_O_2_ reacts mainly with special Cys residues (peroxidatic Cys) within certain proteins, which act as H_2_O_2_ sensors. Second, at room temperature, H_2_O_2_ rapidly equilibrates within a compartment because of its high diffusion rate, and can move between compartments. Even if biomembranes are less permeable to H_2_O_2_ than to water, specific aquaporins transport it across membranes, both in animals and in plants [59,60].

There are roughly 200 different post-translational modifications possible in higher eukaryotes, 60 of which are ROS-dependent, many of which have been detected in plant mitochondrial proteins [61,62]; Cys can undergo oxidation and nitrosylation (also called S-nitrosation); Arg, Lys, Pro, Thr, and Trp can undergo carbonylation; while Trp and Met undergo oxidation. For instance, cysteine thiol groups of proteins are modified under oxidative stress in different ways. Recent studies identified more than 100 sulfenylated proteins in plastids challenged with H_2_O_2_ [63,64]. The Cys residue is oxidized first to sulfenic acid (-SOH), a modification that may be reversed by reduced thioredoxin and glutaredoxin or by reaction with glutathione [65]. Further reaction of sulfenic acid with ROS produces sulfinic (SO_2_H) and sulfonic acid (SO_3_H), which are regarded as irreversible [66]. Around 35 proteins become S-nitrosylated (the -SH group reacts with NO and becomes -SNO) in guard cells after exposure to flg22 [67,68]. Tyr nitration has been detected for many proteins and it is involved in the regulation of their activity [69,70]. Tyr nitration is due first to the interaction of Tyr residues with strong oxidizing agents (hydroxyl and carbonate radicals) derived mainly from peroxynitrite, to generate a tyrosyl radical. These can subsequently react with nitrogen dioxide radical, also deriving from peroxynitrite, to form nitrated Tyr residues. There is a physiological level of Tyr nitration that is intensified upon stress [68,69]. Nitration usually inactivates the protein, but it may also affect signal transduction by competing with Tyr phosphorylation. Another direct effect of NO is the nitrosation of the transition metals (Fe, Cu, and Zn) of the protein prosthetic groups.

Each time a ROS modifies proteins and other molecules, thus affecting their activities, it indeed generates a signal. In those cases, the signal is the produced modification (i.e., Tyr nitration or Cys oxidation to sulfenic or sulfinic acid), as such a modification might change the enzymatic activity of the modified protein. If the activity is inhibited, catalysis will slow down, leading to an increase in substrate(s) and a decrease in product(s). Many enzymes, especially in central metabolism, have been shown to be inhibited by oxidative modifications [8,53,69,70,71]. In other cases, the ROS molecule itself will be the signal, because it can diffuse from one compartment to the other and hence modify the redox status in compartments distant from the point where that ROS have been generated. H_2_O_2_ as well as NO and peroxynitrite, for instance, fall in this category. Interestingly, feed-back effects are foreseeable, as also some enzymes involved in ROS metabolism are affected by ROS, altering their scavenging capacity. For instance, Tyr nitration by peroxynitrite affects the activity of some SOD isoforms in both plants and animals [72], implying that it tends to boost its own production.

### 2.3. Redox State Communication among Compartments

The redox status of one compartment is also reverberated in other compartments by means of simple metabolite shuttles. For instance, cytosol, mitochondria, plastids, and peroxisomes share reducing equivalents through the malate–oxaloacetate shuttle. Triose-phosphate (TP)/3-phosphogylcerate (PGA) allows additional exchange of reducing equivalents between the plastid and cytosol in plants, while, in animals, the glycerol phosphate shuttle involving glycerol 3-phosphate and dihydroxyacetone phosphate allows transfer of reducing equivalents from the cytosol to mitochondria [73]. The development of genetically encoded in vivo redox sensors (which are discussed more in detail in Section 3, together with some other sensors) allow visualization of such movements. For instance, recent studies with NADH sensors [74,75] reveal that (i) there are large variations in the NADH/NAD^+^ ratio among tissues and conditions (for instance, upon sugar supplementation, elicitor exposure, and illumination of seedlings/leaves); and (ii) inhibiting plastidial or mitochondrial ETCs is quite readily reverberated in a change of the ratio in the cytosol, either directly through external NADH dehydrogenases or indirectly through the various metabolite shuttles. A parallel analysis of cytosolic NADH/NAD^+^, ATP, and pH revealed that there is a rapid transfer of reducing equivalents and protons, but not of ATP, from the chloroplast to cytosol upon illumination [76]. Altering the capacity of the malate valves by knocking out the mitochondrial malate dehydrogenases (mMDH1 or mMDH2), or the NADP^+^-dependent plastidic isoform (cpNADP-MDH), did not change the capacity of exporting reducing equivalents or its kinetics in the light. However, the cytosolic NAD pool was more reduced in the dark in all three mutants [76]. It is easy to understand how a reduction in capacity for malate oxidation in mitochondria may lead to a higher NADH accumulation in the cytosol. That the same effect is obtained through a reduction in the NADP^+^-dependent plastidic isoform is more difficult to interpret, but it could mean that a possible increase in NADPH in plastids is relayed to NADH more efficiently in the cytosol via other shuttles.

Thus, altering the redox status by mutation in one compartment can change the balance in other compartments. This is exemplified by the *Mosaic Death 1* (MOD1) mutant of *Arabidopsis*. MOD1 is an enoyl-acyl carrier protein (ACP) reductase involved in plastidial fatty acid synthesis. This enzyme consumes NADH and its absence triggers cell death by increasing the export of reducing equivalents from the chloroplast to mitochondria, mediated by the malate shuttle [77]. Indeed, secondary mutations in malate oxidation in mitochondria or NAD^+^ import into mitochondria suppress the MOD1 phenotype [77,78,79]. Another example is the *gr1* mutant of *Arabidopsis*, impaired in cytosolic glutathione reductase 1. The mutant shows a more oxidized status in the cytosol, which was reverberated in the mitochondria, as measured through cytosolic and mitochondrial redox sensors (roGFP2-Orp1).

## 3. Analytical Techniques to Monitor In Vivo Ca^2+^ and Redox Signaling in Plants: Past and Present Research 

In almost every organism, increases in cytosolic Ca^2+^ concentration ([Ca^2+^]_cyt_) work as a key component in different signal transduction pathways. Depending on the stimulus, cytosolic Ca^2+^ rises can display the form of a single transient or repetitive Ca^2+^ oscillations and are commonly designated as “Ca^2+^ signatures” [80,81,82,83,84]. Generation and shaping of cytosolic Ca^2+^ signatures depend on fine-tuning of the Ca^2+^ influxes and effluxes occurring at both the plasma membrane (PM) and membranes of the different subcellular compartments. In fact, not only cytosol but also the other subcellular compartments (e.g., chloroplasts, mitochondria, endoplasmic reticulum, etc.) experience Ca^2+^ transients, hence putatively participating in the cellular Ca^2+^ homeodynamics and potentially in the shaping of the Ca^2+^ signature, thus ultimately modulating the signal transduction pathways (reviewed [85,86]). Historically, there have been a few elected cellular systems where scientists have investigated the role of Ca^2+^ in signaling processes. Because of the fundamental function in plant drought-stress responses, stomata guard cells represent one of the best model systems from where to investigate the roles of Ca^2+^ signaling [87]. We do cite guard cells because in this system it emerged that, aside from Ca^2+^, other actors were involved in the ABA-induced stomatal closure, with a prominent role played by ROS (i.e., H_2_O_2_) and by their crosstalk with Ca^2+^ [88,89,90]. In this scenario, a milestone discovery was the identification of H_2_O_2_-activated Ca^2+^ currents in the PM of *Arabidopsis thaliana* guard cells, which is responsible for cytosolic Ca^2+^ elevations that contribute to the activation of the anion efflux channels required for the ABA-induced stomatal closure [90,91,92]. Indeed, an impaired activation of these currents in response to ABA strongly reduces the stomata closure, thus affecting the plant’s fitness in drought conditions [93,94]. Surprisingly, even though this discovery was made 20 years ago, the molecular identity of the H_2_O_2_-activated Ca^2+^-permeable channel(s) is still unknown. For this reason, their genetic identification is of utmost importance to decipher the mechanisms involved in plant responses to a changing environment. Nevertheless, further research demonstrated that Ca^2+^ and H_2_O_2_ crosstalk reactions occur in different cell types and at different levels with several feedback mechanisms and self-amplifying loops [95]. One of the most striking evidence pieces in support of this is offered by plant NADPH oxidases, known as respiratory burst oxidase homologues (RBOHs) [96,97]. RBOHs are PM-localized enzymes that produce in the apoplast superoxide anions that are then converted to H_2_O_2_ by dismutation [97]. Notably, through RBOHs, ABA induces in guard cells the production of H_2_O_2_, thus activating Ca^2+^ currents across the PM [90]. A very recent discovery ascertained that the H_2_O_2_ activation of the Ca^2+^ currents depends on the activity of the H_2_O_2_ receptor HPCA1, a member of the leucine-rich-repeat receptor kinase family [98]. An interesting feature of RBOHs is that their activity is boosted by Ca^2+^ through direct interaction with Ca^2+^ (through EF hands) or by Ca^2+^-dependent phosphorylation [99,100]. So, a self-amplifying Ca^2+^–H_2_O_2_ loop exists in guard cells. Noteworthily, RBOHs are also regulated by other mechanisms, including cysteine persulfidation (H_2_S). A recent work demonstrated that ABA stimulates, through the activity of L-cysteine desulfhydrase 1, the persulfidation of the RBOHD at Cys825 and Cys890, enhancing its ability to produce ROS, thus positively regulating the ABA-induced stomatal closure [101]. 

Whereas guard cells represent a peculiar cell system, there is a wealth of examples clearly reporting the existence of a Ca^2+^–ROS crosstalk in other cell types and in response to different environmental challenges. In particular, in leaf tissues, cells respond to pathogen attack with a quick production of ROS by NADPH oxidases [100]. In this interaction, key players are the pathogen-associated molecular patterns (PAMPs), which are, for example, released by invading bacteria (e.g., flg22 from *Pseudomonas syringae*) and recognized by pattern recognition receptors (PRRs) [100]. Interestingly, the PRRs’ binding of bacterial PAMPs triggers, at first, a cytosolic Ca^2+^ increase, followed by the rapid and strong production of ROS [100]. Of note is the demonstration that such a PAMP-induced ROS production is also dependent on RBOHD activity, which, as reported above, is the same isoform involved in the ABA-induced stomatal closure [88]. So, pioneer studies carried out in guard cells have revealed the existence of key basic mechanisms that are shared with other cell types. Indeed, the NADPH oxidase-dependent Ca^2+^–ROS crosstalk is a model that has been predicted to play a pivotal role also in developmental programs such as pollen tubes and root hair growth [102,103,104,105,106]. The Ca^2+^–ROS crosstalk model has been predicted to sustain and permit long-distance signaling that occur in plants in response to biotic and abiotic stresses [107,108,109,110]. Systemic wound responses (SWRs) and systemic acquired acclimation (SAA) to abiotic stress are triggered by rapid waves of Ca^2+^ and H_2_O_2_ traveling over a long distance (from leaf to leaf and from leaf to flowers) [95,110,111]. One of the plausible models working in long-distance signaling is therefore represented by a repetition of the Ca^2+^–H_2_O_2_ module in adjacent cells, where the H_2_O_2_ produced by a cell can trigger a cytosolic Ca^2+^ increase in the neighboring cell, which, in turn, stimulates the synthesis of H_2_O_2_ via RBOHs in a self-amplifying loop [95,110,112]. Whether this model may be active also during developmental processes, such as embryo development and differentiation, is not currently known. However, it is interesting to note that, in *Arabidopsis thaliana*, the glutamate receptor-like AtGLR3.5 (a predicted Ca^2+^-permeable channel) is expressed in germinating seeds and is involved in the regulation of the cytosolic Ca^2+^ concentration that counteracts the effect of ABA to promote germination [113]. Moreover, GLR3.1 and GLR3.5 in guard cells are predicted to maintain the basal cytosolic Ca^2+^ levels required for RBOH activity [114]. Interestingly, ROS have been reported to play a pivotal role in the regulation of seed germination and dormancy but how they exert their roles is far from being understood (discussed later, reviewed in [115])

Monitoring Ca^2+^/H_2_O_2_ crosstalk during seed development and germination could be an interesting aspect to study, for instance, to understand the role of these two second messengers during embryo and seed development. In this light, powerful tools like the use of emerging modern imaging technologies are providing new insights in the field. As a proof of concept [116], by using a fluorescence plate reader and *Arabidopsis* plants expressing MgATP^2−^, a team sensor demonstrated that seeds, within minutes after imbibition, rapidly accumulated ATP [117]. This piece of evidence paves the way to perform similar experiments to look at Ca^2+^ and H_2_O_2_ in intact seeds. It must be said that since the H_2_O_2_ production might be transient, and that it can exert its role through the modification of the cysteine residues of the target proteins [118], one could also consider monitoring the redox status of antioxidant pools, such as glutathione.

**Figure 2:**
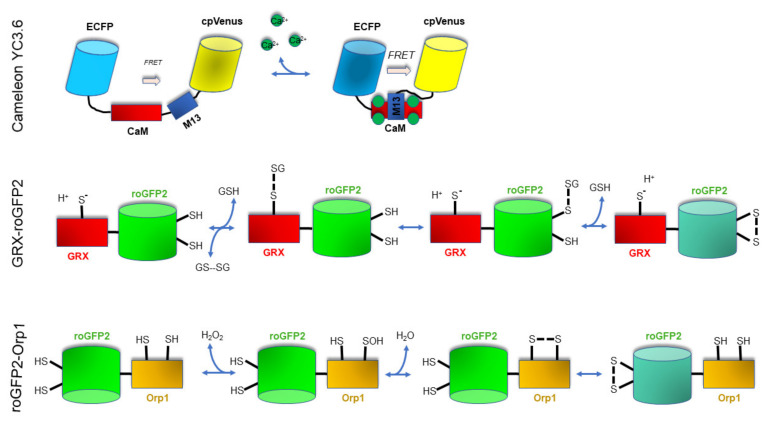
Schematic structure of a Ca^2+^ sensor (Cameleon YC3.6, (**top**)), a redox sensor (GRX-roGFP2, (**middle**)), and H_2_O_2_ sensor (roGFP2-Orp1, (**bottom**)). Ca^2+^ binding to the CaM domain rearranges the structure and brings the ECFP close to the cpVenus, thereby augmenting the efficiency of FRET between the two fluorescent protein domains. In the redox sensor, the oxidized glutathione dimers, accumulating during oxidative stress, react with a sensitive Cys residue of the GRX, forming a disulfide bond. The bond is transferred to the roGFP2 moiety (right) and eventually affects fluorescence emission. When reduced GSH prevails, the sensor is brought back to the reduced form. The ROS sensor reacts with H_2_O_2_ (left), forming a sulfenic group. This undergoes dehydration, leading to disulfide bridge formation, which is transferred to the roGFP2 moiety (right), altering fluorescence emission. Dashed lines between the sulfur atoms represent disulfide bonds.

To precisely study in vivo and non-invasively the levels of Ca^2+^, H_2_O_2_, and the redox status of the cellular components, we suggest the use of genetically encoded sensors, and in particular ratiometric ones. This suggestion is primarily based on the assumption that the readout of ratiometric sensors is strongly independent of their amount in the cell. This property can also be important when samples are imaged by microscopy, since it allows the correction of focus changes or moving artefacts. For these reasons, we feel comfortable to propose the use of ratiometric genetically encoded sensors for the measurement of Ca^2+^, H_2_O_2_, and the redox status of GSH. There are different genetically encoded ratiometric sensors for the in vivo measurement of these parameters (for this reason, we redirect the interested readers to recent reviews [86,119,120]). However, here we review some tools that are currently used in our lab and that could be used for reliable analyses during embryo and seed development. A schematic representation of their structure and function is presented in Figure 2.

### 3.1. Imaging Techniques to Monitor In Vivo Ca^2+^ Levels and Dynamics 

Based on our direct experience, the Yellow Cameleon YC3.6 [121] represents an excellent tool for measuring in vivo Ca^2+^ levels and dynamics in entire organs and tissues [122,123,124,125,126,127]. Specifically, the development of the YC3.6 has greatly advanced the ability to analyze Ca^2+^ dynamics in vivo with an unprecedented spatial and temporal resolution [128]. YC3.6 is a FRET-based indicator protein. The FRET phenomenon consists of an excitation energy exchange between two fluorescent molecules, an acceptor and a donor, whose absorption spectra partially overlap. This energy transfer occurs when the two molecules are at distances that vary between 2 and 10 nm. In the YC3.6 biosensor, two green fluorescent protein (GFP) variants, cyan fluorescent protein (CFP) and the circularly permuted Venus (cpVenus), are linked together by the Ca^2+^-binding protein calmodulin and the calmodulin-binding peptide M13 [129]. Binding of Ca^2+^ to the calmodulin of YC leads to a conformational change in the indicator, bringing CFP and cpVenus into proximity and allowing an enhanced FRET. The efficiency of FRET allows quantitative measurements of the [Ca^2+^] variations to be made by recording the ratio shifts along a time-course [128]. The higher the Ca^2+^ concentration, the higher the cpVenus/CFP ratio. Traditional wide-field and confocal fluorescence microscopes able to specifically excite CFP and collect the emissions from the CFP and cpVenus can be efficiently used to measure the CFP/cpVenus FRET, as well as a fluorescent plate reader [126,130]. *Arabidopsis thaliana* lines expressing the Cameleon YC3.6 targeted to cytosol as well as to different subcellular compartments are available and can be used in entire organs [131] (reviewed in [86,132]). 

The imaging of Ca^2+^ in plants essentially adopts techniques developed for analyses in animal cells; therefore, the use of Cameleon has been largely exploited to monitor the Ca^2+^ levels and dynamics in the cytosol, nucleus, ER, Golgi apparatus, or endosomes of single living cells ([133] and references therein).

### 3.2. Imaging Techniques to Monitor In Vivo the 2GSH/GSSG Redox Status 

Biochemical techniques have been largely used to study the redox status of the most important antioxidant pools or to study the activity of ROS scavenging enzymes. However, in most cases the biochemical assays require sample destruction and tissue homogenization, which dramatically reduces the sensitivity of the analysis and can also introduce artefacts due to sample manipulation [118,119]. To overcome these issues, in the last 15 years, biologists started to use new in vivo technologies that rely on the use of genetically encoded sensors. This enables real-time monitoring of thiol redox dynamics and possibly of the production of specific ROS.

The most used redox genetically encoded sensors in plants are roGFP1 and roGFP2. These two sensors are modified GFP where two cysteine residues have been inserted in adjacent β-strands on the surface of the protein β-barrel [119,134,135]. This modification renders the proteins sensitive to a change in the cellular redox potential; in fact, the cysteine residues being positioned near the chromophore, they can form a disulfide bond that leads to a structural change that influences protein fluorescence. Similar to Cameleon, roGFPs are ratiometric sensors, since the disulfide bond formation or its breaking changes the quantum yields (QY) of the two main absorption peaks (at 405 and 488 nm, respectively) in an opposite way, leading to a ratiometric response [136]. The higher the oxidation status (hence the disulfide bridges formation), the higher the 405/488 nm ratio. Based on extensive research carried out in animals and plant cells, it has been clearly demonstrated that roGFPs are in a redox potential equilibrium with the 2GSH/GSSG couple in a reaction catalyzed by glutaredoxin (Grx) (reviewed in [119]). Both the GSH pool and Grx are present in the cytosol and in the subcellular compartments [119]; however, since in vivo the roGFPs equilibrium with the 2GSH/GSSG couple is mediated by Grxs, it may be assumed that the roGFPs oxidation/reduction is limited by the availability of endogenous Grxs. To overcome this problem, the human Grx1 was fused to the roGFP2, making a 1:1 proportion between the probe and the catalyst, hence de facto eliminating any possible issue [137]. *Arabidopsis* lines expressing the Grx1–roGFP2 targeted to cytosol as well as different subcellular compartments are available and can be used in entire organs [126,136]. 

As in plants, both chemically and genetically encoded probes are available for the detection of redox-mediated signaling and oxidative stress in animal cells and organisms. Advancements in imaging techniques and available probes have been intensively reviewed [138,139,140,141]. Importantly, transgenic animals—both “lower” vertebrates’ models, such as zebrafish, and mice, stably expressing tissue-specific, genetically encoded probes, such as roGFP—are being used to address important biomedical questions (for example, see [139,142]). 

### 3.3. Imaging Techniques to Monitor In Vivo H_2_O_2_ Levels and Dynamics

Some peroxidases have an intrinsic and powerful capacity to act as H_2_O_2_-dependent protein thiol oxidases when they are recruited into proximity of oxidizable target proteins [137]. Hence, the Yeast Orp1 peroxidase was fused to the roGFP2 to generate an H_2_O_2_-dependent roGFP2 oxidation sensor: the roGFP2–-Orp1 [137]. The way roGFP2–Orp1 is used is the same as that one of roGFP2 and Grx1–roGFP2, but the difference is that the oxidation is driven primarily by H_2_O_2_ and not by other oxidants. Nevertheless, an important aspect of roGFP2–Orp1 is that its oxidized form is reversibly reduced in vivo, most probably by the Grx and/or the Trx system that directly reduces Orp1 [74]. Because roGFP2–Orp1 undergoes a specific H_2_O_2_-dependent oxidation, but can also be reduced, its redox state is influenced not only by the level of the oxidant (H_2_O_2_) but also by the reductants themselves, such as GSH and thioredoxin. This makes the use of this probe not completely straightforward for the definition of the H_2_O_2_ levels [119]. However, roGFP2-Orp1 has been successfully expressed in *Arabidopsis* plants, allowing to monitor in the cytosol and mitochondria the intracellular H_2_O_2_ dynamics in response to exposure to elicitors such as flg22 and chitosan [74]. 

A new promising H_2_O_2_ ratiometric sensor is the genetically encoded fluorescent protein HyPer7 [143]. HyPer7 is a modified fluorescent sensor composed of a circularly permuted GFP integrated into the OxyR domain from *Neisseria meningitis*, which is sensitive to very low H_2_O_2_ concentrations. This allowed the detection of subtle changes in H_2_O_2_ concentrations in the cytosol and mitochondria of animal cells [143], but it also might represent a new useful tool for similar studies in plants.

## 4. ROS/Ca^2+^ and the Impact in Differentiation and Developmental Processes in Plants

ROS are end products of multiple and fundamental cellular processes, such as aerobic respiration and photosynthesis, for plant life. As previously described, ROS play key roles in the physiological reprogramming required for plant development [144]. ROS, for instance, act as potent oxidants affecting cell wall cross-linking and extensibility [145,146,147,148], as well as signaling molecules controlling a wide range of biological processes [12,149,150,151,152,153,154]. 

In response to biotic and abiotic stresses, after mechanical wounding and during developmental processes, ROS are generated by reduction of O_2_ [155]. At the subcellular level, ROS are produced in several organelles (e.g., chloroplasts, peroxisomes, and mitochondria), but ROS also can be produced in plant cell walls and plasma membranes [7,148,156]. The cell wall is fundamental for the fine control of developmental processes and also to activate specific responses to biotic and abiotic stresses [157,158,159,160]. Indeed, the existence of enzymatic machinery specifically devoted to produce ROS in the cell wall suggests the importance of spatially and temporally regulated ROS production for the control of differentiation processes and, importantly, to trigger fast, local, and active defense mechanisms in response to mechanical injury [148,161,162,163]. In the following section, we describe some of the recent advances uncovering the role of ROS in signal transduction affecting plant developmental processes (see Table 1). 

### 4.1. Seeds, ROS, and Germination

The ability of seeds to survive in harsh environments preserves their longevity. Seeds eventually germinate when the environmental conditions favor the survival and the thriving of the emerging seedlings. These are certainly among the most fascinating and challenging attributes of seed plants [191]. The germination process, which is characterized by rupture of the integument, initial protrusion of the radicle, and the elongation of the embryonic axis, is certainly triggered by favorable conditions of light, temperature, and humidity; however, these conditions might be not sufficient to trigger seed germination in dormant seeds. Dormancy is indeed a protective mechanism aimed at preventing the pre-harvest sprouting; dormancy also prevents simultaneous germination of all the seeds and the competition among them that such a situation would imply [192,193,194]. Interestingly, ROS take part both in dormancy and germination processes [195,196]; they are produced during the drying of the seeds, as well as during imbibition, and represent key players in both seed dormancy and longevity [195,197]. In particular, seeds can germinate when the ROS levels are within a given range; such a range is known as the “ROS concentration window”, and below that concentration seeds would not germinate, whereas above that concentration seeds would suffer from the toxic effects of ROS and their longevity would be compromised [197,198,199]. The existence of a “ROS oxidative window”, needed for seed germination to occur, has been demonstrated by exogenous application of oxidants, by pharmacological or genetic means [12]. For example, *A. thaliana* plants overexpressing thylakoidal ascorbate peroxidase, tAPX, which scavenges H_2_O_2_ (with ascorbate as electron donor), show increased resistance to paraquat-induced photooxidative stress and to nitric oxide-induced cell death; seeds of the transgenic *A. thaliana* line with the highest *tAPX* overexpression germinated later than the control seeds and also later than other *tAPX* transgenic lines with moderate overexpression [200]. However, at that time, the connection between the ROS levels and dormancy went unnoticed. Nonetheless, these results, revisited in light of the “ROS oxidative window”, would suggest that ROS depletion in *tAPX* plants might also affect developing seeds, and hence, in turn, also their dormancy, which was prolonged in the *tAPX* overexpressing line. This shift in the “ROS oxidative window” would indeed explain the enhanced longevity observed in 26–30 months after-ripened *tAPX* seeds, when compared to their wild type line [201]. More recently, measurements of the H_2_O_2_ levels in barley seed embryos after imbibition showed higher values in non-dormant seeds than in dormant seeds [202]. These authors reported that activity of NADPH oxidase (producing ROS) and of catalase were, respectively, lower and higher in non-dormant seeds than in dormant ones [202]. The action of H_2_O_2_ during germination is multifaceted and it involves endosperm loosening and oxidation of the reserve proteins as well as of the mRNAs, together with the signaling crosstalk with ABA and GA, the two hormones acting in an antagonistic way in the regulation of the germination process, and nitric oxide (NO) [12,203,204,205]. It should be underlined that the ROS status of the cells are dependent on their Fe status, and in particular, on the concentration of uncomplexed, redox-active Fe ions, due to their role in catalyzing the Haber–Weiss reaction [206]; indeed, the Fe nutritional status of a mother plant also affects the dormancy of progeny seeds [191]. 

### 4.2. ROS Fine Tune Control on Stem and Root Differentiation in Plants 

ROS have multiple roles in plant stem cell regulation, as several works have shown dynamic changes in the expression pattern of ROS biosynthetic enzymes during stem cell differentiation [207]. As elegantly demonstrated by Zeng and collaborators, the fine-tuned balance between superoxide and H_2_O_2_ works as a key developmental activator that allows the transition from stem cell maintenance into stem cell differentiation by antagonistically regulating the expression of the transcription factor WUSCHEL [207]. H_2_O_2_ is accumulated specifically in the differentiating peripheral region in order to promote stem cell differentiation and antagonistically regulate superoxide accumulation in stem cells by inhibiting key enzymes in superoxide metabolism. The differentiation program involves the repression of SOD and the concomitant activation of peroxidases, establishing the high superoxide/low H_2_O_2_ ratio in plant stem cells. During the last years, multiple studies have demonstrated the fundamental role of ROS in root differentiation [8,166,168,208]. The superoxide^/^H_2_O_2_ ratio varies along the roots: while the concentration of superoxide decreases gradually from the meristem to the transition zone, the concentration of H_2_O_2_ decreases gradually from the differentiation zone to the elongation zone in the root [209,210]. This balance has been shown to be controlled by UPBEAT1, a basic helix–loop–helix (bHLH) transcription factor, which regulates the expression of a set of peroxidases that control the ROS gradient in the root meristem [168,210]. The transcriptional cascade controlling ROS during root differentiation has been further investigated, demonstrating that hormones such as brassinosteroids define root meristem activity by controlling the ROS balance [211]. Multiple transcriptional factors, such as APP1, ABO8, PHB3, and RITF1, are involved in the brassinolide signaling pathway that controls the ROS signal regulating root stem cell maintenance [166]. Modulation of ROS homeostasis, and its possible involvement in hormone regulation via IAA degradation, has been recently reported to control meristem maintenance in roots [8]. A connection between ROS balance and other hormones, such as brassinosteroids, in the control of root meristem also has been highlighted recently [211].

### 4.3. ROS Are Crucial in Different Steps during Sexual Plant Reproduction

The process of pollination, which is fundamental for sexual reproduction in almost all plants, has provided excellent examples on how ROS regulation controls developmental processes [1,212]. Different developmental steps are required for successful pollination in plants [213]. Once pollen grains reach the stigma, the pollen adheres and hydrates, and then germinates on it, creating an elongating pollen tube. The pollen tubes grow over a long distance (for instance in maize it can be more than 30 cm) from the stigma toward the ovary in order to reach the female gametophyte. Upon arrival at the female gametophyte, the pollen tube releases two sperms in a degenerated synergid cell for fertilization. Throughout this process, there are continuous interactions, mediated by ROS, between the pollen structure, maternal tissues (stigma, style, and ovule), and female gametophyte, which are necessary for proper pollen hydration and germination, pollen tube growth through the transmitting tract of the pistil, guidance to the ovule, reception of the female gametophyte, and sperm–egg cell fusion (see the recent review by [106]).

**Figure 3:**
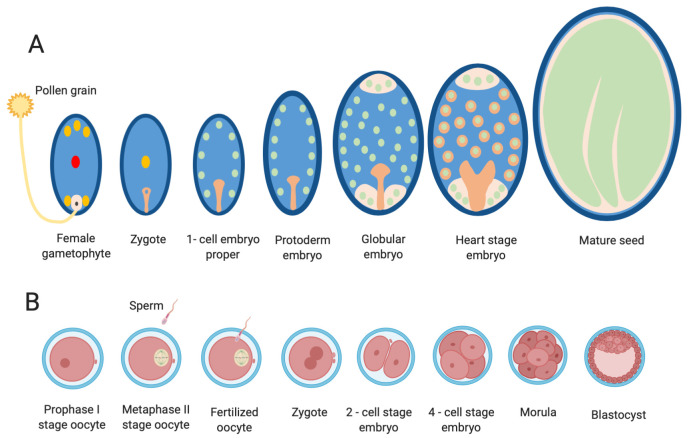
Schematic representation of fertilization and embryonic development in flowering plants (**A**) and mammals (**B**). Created with BioRender.com (https://biorender.com/, accessed on 28 January 2021). (**A**) In angiosperms, once the pollen grain attaches on the tip of the pistil, the pollen tube grows through the pistil and reaches the ovule located in the ovary. Upon fertilization, the ovule transforms into a seed. The ovule contains the female gametophyte, which consists of different specific cell types: 1 egg cell, 1 central cell, 2 synergid cells, and 3 antipodal cells. The two synergid cells attract the pollen tube in the direction of the ovule. When the pollen tube reaches the interior of the female gametophyte (embryo sac), the pollen tube delivers the two sperm cells inside the ovule. One of the sperm cells fuses with the egg cell, forming a diploid embryo, while the other sperm cell fuses with the nuclei of the central cell, forming a triploid endosperm. Once the zygote has been constituted, it undergoes a first asymmetric cell division. This mass of cells that constitute the embryo will be visible after several days of active division. The structures created in the process of double fertilization generate thus a diploid embryo and a triploid endosperm. The endosperm tissue surrounds and nourishes the embryo and it is typical in angiosperm seeds. In some species, it is completely absorbed at maturity (non-persistent endosperm) while, in others, like in most of the cereals, the endosperm is present until germination (persistent endosperm), and the cotyledons serve to absorb the stored nutrients delivered from the endosperm upon germination. At maturity, the maternal seed coat fully develops around the embryo and the endosperm. At the end of the process, a mature seed is formed containing a protected embryo that, after germination, can develop into a young plant. (**B**) In animals, the Prophase I arrested oocyte undergoes a process called oocyte maturation during which the oocyte completes meiosis I, extrudes the first polar body, and progresses to the metaphase stage of meiosis II. The fertilizing sperm penetrates the MII stage oocyte and forms the paternal pronucleus. The oocyte, in turn, completes meiosis II, extruding the second polar body and forming the maternal pronucleus. After DNA replication, the maternal and paternal genomes reconstitute the diploid status in a process called syngamy, the chromosome condense, and the first mitotic division of the zygote occurs. Thereafter, the blastomeres undergo consecutive mitotic divisions forming the blastocyst. In most mammals, oocyte maturation occurs in the ovarian follicle, while fertilization and early embryonic development occur in the oviduct. Afterwards, the embryo reaches the uterus where implantation occurs.

We will first review the current literature on ROS and Ca^2+^ signal transduction in sexual reproduction in flowering plants and animals (see later). Although they constitute different biological systems, embryogenesis in plants and animals has common aspects, such as the male–female gamete interaction and subsequent events of cell proliferation, differentiation, morphogenesis, and embryo development in a maternal-containing environment (see schematic Figure 3 for a compared developmental schematic overview of embryogenesis in plants and mammals). 

In most angiosperms, the female gametophyte develops within the ovule and is formed by two antipodal cells, one central cell, two synergid cells, and one egg cell. The cells present in the embryo sac are highly polarized. For instance, while the egg cell nucleus is located toward the chalazal end of the embryo sac, the synergid and central cells have the opposite localization. During gametophyte formation, ROS levels are finely controlled, and they vary depending on the gametophyte cell type. For instance, it has been reported that superoxide and peroxide levels are present in central cells but not in antipodal cells, which later undergo cell death in mature embryo sacs [175]. The central cell has been reported to regulate antipodal cells lifespan. Notably, the accumulation of ROS in the central cell mitochondria might work as a signal to trigger antipodal cell death in a non-cell autonomous way [175]. ROS generated by mitochondria have an important role in regulating cell fate and embryo sac polarity [175]. In *Arabidopsis*, several studies focused on the role of ROS in female gametophyte development, for instance, via SOD [156]. Most plants have different types of SOD: iron SOD (FeSOD), manganese SOD (MnSOD), and copper–zinc SOD (Cu/ZnSOD). This is considered the first line of cellular defense against superoxide. Its importance was demonstrated, for instance, by the characterization of the *Arabidopsis oiwa* mutant, a female gametophytic mutant impaired in mitochondrial manganese SOD (MSD1). In this mutant, high levels of peroxide and mitochondrial superoxide accumulate in the central cell as well as the micropylar cells. Interestingly, a range of female gametophyte phenotypes accompanied these alterations; for instance, the mitotic arrest during megagametogenesis or the partial central cell identity determination [175]. Another example is the developmental characterization of the *athemn1* mutant defective in tetrapyrrole biosynthesis, which accumulates more ROS in developing embryo sacs and has defects in female gametophyte development, with embryo sacs displaying unfused polar nuclei [176]. Interestingly, central cell differentiation is impaired in the *athemn1* mutant. Upon fertilization, its seeds present defective endosperm development and embryo developmental arrest [176]. The role of mitochondrial ROS for proper embryo sac development in female gametophyte development has been demonstrated in several works. This link has been shown in multiple *Arabidopsis* mutants and excellent reviews are available [214]. Interestingly, a potential role of oxidative stress in the balance between sexual megasporogenesis and apomixis program in facultative apomictic plants also has been proposed recently [215,216]. Further development of research in this context could provide interesting clues in order to understand how environmental factors influence apomixis via ROS-. This has a major impact for breeding programs aiming at introducing apomictic traits to avoid genetic variability associated with sexual reproduction.

However, the role of many players affecting ROS in each cell has yet to be discovered. Single-cell transcriptome data are now available for specific plant structures, such as the female gametophyte [217]. One can thus explore the variation in the level of specific transcripts involved in ROS metabolism among cell types in female gametophyte development. In order to provide candidates for future research, we analyzed this dataset (see Table 2). Interestingly, there are multiple members of genes involved in ROS metabolism, such as SODs, peroxidases, glutathione peroxidases, ascorbate oxidases, and glutathione peroxidases, which are differentially expressed in specific female gametophyte organs such as synergids, egg cells, or the central cells. We found very few genes substantially expressed in the synergids, especially at a high level (e.g., *Catalase1*). In the central cell, some genes (*Catalase1*, *Glutathione Peroxidase 2* and *6*) were expressed at a high level and many at an intermediate level (*Glutathione Peroxidase1*, *3*, and *8*). An intermediate situation is taking place for the egg cell, where there are fewer genes expressed compared to the central cell, but usually at a moderate level. Some genes represent interesting candidates involving the cross talk among the cell types and the tissues derived from them (embryo/endosperm). For instance, *Glutathione Peroxidase 2* seems to be expressed solely in the central cell, while isoform 6 is apparently expressed in all three cell types, although at different levels. For example, looking at the genes of the canonical VitC biosynthetic pathway, AT3G02870 (*VTC4*) is apparently expressed in both the egg cell and central cell, while AT5G55120 (*VTC5*) and AT3G47930 (*GalLDH*) are expressed only—or almost exclusively—in the central cell. It might be worth looking at the developmental defects in preliminary developmental steps around fertilization or at early embryo development in the respective mutants. The hypothetical altered enzymatic activities of genes that act as scavengers (or that are involved in the production of scavengers) of ROS are likely to locally affect the ROS levels in specific cell types in which they are expressed, and might impact primarily the functionality of these cells. Future advances will provide knowledge on the role of those specific ROS-related regulators in plant reproduction.

The role of ROS in mature female gametophyte function has also been investigated in depth during the last years and there is plenty of literature. Upon pollination, pollen grains land on the stigma of a flower. After crossing the stigmatic barrier, the pollen tube begins to grow through the style and the transmitting tract and it navigates through the funiculus, the structure that attaches the ovule to the placenta. At this stage the pollen tube exits out of the intercellular space and grows along the funiculus, reaching for the micropylar aperture of the ovule. The pollen tube enters the ovule through the micropylar region and bursts to release two sperm cells into the female gametophyte. The double fertilization in angiosperms thus requires two sexual fusions; one sperm fuses with the egg cell and leads to the diploid embryo formation, the other one fuses with the central cell, forming the triploid endosperm. The role of ROS in pollen tube growth and pollen-pistil interaction has been excellently reviewed in detail [106]. One of the key functions performed by ROS in the female gametophyte takes place during pollen tube–synergid cell interaction. Synergid cell death is critical in three key steps of the proper angiosperm fertilization process [218]. First, synergid cell death is necessary for pollen tube entry into the ovary. Secondly, the degenerated state of synergid cells is required for tube growth cessation and release of pollen tube contents. Third, synergid degeneration accompanies the cytoskeletal reorganization necessary to facilitate migration of the two sperm cells to the egg and central cells. Thus, the synergid cells are necessary for successful fertilization by communicating with the pollen tube. They secrete small cysteine-rich peptides LUREs in order to guide the pollen tube growth to reach the ovule [219,220,221]. Pollen tube receptors sense LURE peptides secreted from the synergid cells to orientate pollen tube growth into the ovules. After being attracted to the ovule, signals from the synergid are again perceived by the pollen tube. These signals trigger changes in the pollen tube tip, leading to pollen tube burst and delivery of the sperm cells to the female gamete [221]. It has been proposed that an oxidative environment at the female gametophyte is required for pollen tube reception at the synergids [175]. H_2_O_2_ was detected in a specific temporal window specifically in synergid cells; after pollen arrival onto the stigma but before pollen tubes reach the ovule [175]. Supporting the importance of ROS in pollen tube attraction, high ROS levels were detected in the ovule micropyle, the specific region that allows pollen tube penetration into the ovule [222]. Chemical application of ROS scavengers to excised pistils to highlight the role of ROS in pollen tube attraction was performed by Duan and collaborators. They found that pollen tubes were attracted to ovules but they continued to grow and failed to burst and release the sperm, thus confirming that local ROS changes are critical for proper pollen timing on the tube attraction necessary for double fertilization [222]. Consistent with these inhibitors assays, recent evidence supports the role of ROS in the process by studying the synergid-specific receptor-like kinases (RLKs) involved in pollen tube reception: FERONIA (FER), HERKULES1 (HERK1), and ANJEA (ANJ) [180]. It was recently shown that *fer* mutants also display a pollen tube overgrowth phenotype and do not accumulate ROS in the micropyles of ovules, providing evidence that micropylar ROS levels is an important determinant controlling pollen tube reception [180]. 

The role of Ca^2+^ signaling in synergid cells during pollen tube reception was also investigated [186,187,223]. Studies performed with the *Arabidopsis* synergid-specific gene NORTIA (NTA) have shown how Ca^2+^ oscillation in synergids is fundamental for proper pollen tube reception. *nta* mutants display a pollen tube overgrowth phenotype [224]. A recent study reported that also the Ca^2+^ signal is required for proper pollen tube sensitivity to ovule signals in *Arabidopsis thaliana*; MLO5 and MLO9 can recruit a Ca^2+^-specific channel at the plasma membrane via SNARE proteins in order to fine-tune and modulate the Ca^2+^ gradients in the pollen tube [186,187]. 

The last step of pollen tube reception involves pollen tube bursting in order to release the sperm cells. Pollen tube discharge triggers synergid degeneration in a coordinated way, involving a pollen tube–synergid interaction [225]. The role of ROS triggering synergid cell degeneration by initiating a programmed cell response (PCD) has been proposed, but these mechanisms are still unknown. The connection between micropylar ROS and synergid control of pollen tube behavior may be more complex than previously thought. Higher resolution imaging of ROS dynamics and Ca^2+^ sensors during pollen tube arrival to the synergid cells in specific mutants will be essential in order to clarify the role of ROS and Ca^2+^-waves during reproduction in plants. The use of specific sensors targeted in different cell types appears as a promising strategy for better understanding the role of ROS in the process (as discussed previously).

Several studies focused on the involvement of ROS in pollen functioning. Upon landing on the stigma, pollen grains undergo adhesion and hydration and trigger a germination program in order to generate the pollen tube [213]. For instance, in *Arabidopsis* it was shown that the NADPH-oxidase homologues, *RbohH* and *RbohJ*, are the source for most ROS produced at the pollen tube apex [103]. The relative double mutant presented low ROS generation and concomitantly exhibits bursting in vitro and delayed growth in the pistil [185]. Those studies suggest that Rboh are critical for proper pollen tube growth and are involved in maintenance of cell-wall integrity [103,185]. It was shown that in the null mutant of *Arabidopsis KINβγ*, a plant-specific subunit of the SNF1-related protein kinase 1 complex, the ROS levels of the pollen grains were reduced and concomitantly pollen adhesion to the stigmatic surface was impaired [226]. Referring to the pollen–pistil interaction, it has been suggested that the ROS signals originating at the pollen grains mediate the interaction with the stigma by controlling the expression of the inward shaker K^+^ channel *SPIK* in pollen [227]. Pollen–stigma signaling is involved in the different mechanisms of self-incompatibility in multiple plant species [214,228]. ROS regulation is important for the control of self-incompatibility processes in plants, as reported in many species, although specific strategies might differ between plant systems and developmental stages, including defense functions, signaling, and senescence [229]. It has been shown that ROS triggers programmed cell death (PCD) in self-incompatible response [230]. More examples of the role of ROS controlling the pollen–pistil interaction was reported recently in ornamental plants such as kale (*Brassica oleracea* var. *acephala*), a self-incompatible species. The decreased ROS levels in kale stigma exposed to exogenous flavonoid treatment had a negative impact on the attachment and successive germination of the compatible pollen. In recent years, multiple works have investigated ROS signaling in pollen–stigma communication—we apologize for not discussing them because of space limitations [212,214,229,231].

## 5. ROS/Ca^2+^ Crosstalk in Mammalian Embryonic Development

### 5.1. Overview of Gametogenesis and Early Embryonic Development in Mammals

In mammals, the female gamete, the oocyte, is the largest cell of the body and has the intrinsic ability to initiate embryonic development once it has undergone a process known as activation, while the paternal gamete, the spermatozoon, has developed the ability for motion and penetration of the oocyte’s investments. A schematic representation of fertilization and early embryonic development is shown in Figure 3B.

Oocyte and sperm development are complex processes that initiate very early during development of a new individual (for comprehensive description, please refer to [232]). Once the primordial germ cells (PGC) migrate to the forming gonad, they develop into oogonia and spermatogonia, which are the pluripotent cells from which the male and the female gametes originate. Oogonia and spermatogonia undergo further proliferation before they enter gametogenesis that in turn includes meiosis and cytodifferentiation. These processes largely occur in the gonads in cooperation with their somatic cell components.

In the female, oogenesis starts in the fetal ovary and terminates only after puberty is reached and fertilization eventually occurs. In the fetal ovary, oogonia differentiate into cells called primary oocytes, which duplicate their DNA and then enter the prophase of meiosis I, when they become arrested at the diplotene stage. Primary oocytes are covered by ovarian somatic cells (called follicular cells), thus forming the primordial follicles, which constitute a pool from which the female will recruit follicles for growth and ovulation for the rest of the reproductive life. Follicular growth occurs when follicles are recruited from the primordial pool (follicle activation) and develop into primary, secondary, and tertiary follicles. Concomitant to follicle activation, the enclosed primary oocytes reactivate RNA synthesis and are engaged in a phase of growth during which a large amount of new molecules are produced and stored in the oocyte cytoplasm. Important morphological and functional changes, such as the synthesis of a protective glycoprotein shell—the zona pellucida—and the development of specialized cytoplasmic organelles, occur during the growth phase. At the end of the growth phase, in the tertiary follicle, transcriptional activity is again globally silenced, and the oocytes, now referred to as “fully grown” oocytes, are ready to resume meiosis. Meiotic resumption, which is cyclically triggered only after puberty by hormonal stimulation, is followed by progression to the metaphase, anaphase, and telophase of meiosis I. Completion of meiosis I is followed by an asymmetric cell division in which half of the homologous chromosomes are extruded in a small cell, called the polar body. Then, the large oocyte enters meiosis II, the chromosomes are arranged in the metaphase II plate, and meiotic progression arrests again until fertilization eventually occurs. Progression from the prophase I to the metaphase II stage is referred to as “oocyte maturation”. In most species, oocyte maturation occurs within the ovarian follicle and the metaphase II stage oocyte is released from the ovarian follicle into the oviduct, the anatomical site of fertilization, by a process called ovulation.

In the male, spermatogenesis begins after puberty and includes three main steps: spermacytogenesis, meiosis, and spermiogenesis. Spermacytogenesis leads to the generation of primary spermatocytes by several mitotic divisions. Primary spermatocytes then undergo and complete meiosis, which produces haploid spermatids. Spermiogenesis follows the meiotic division and includes a series of morphological and functional changes that ultimately re-structure the round-shaped spermatids into differentiated spermatozoa. Final functional maturation of the spermatozoa, including acquisition of motility, capacitation, and acrosomal reaction, occur in the male and female genital tract.

When the fertilizing sperm penetrates the oocyte, the maternal and paternal genomes are united in a single cell: the fertilized ovum, forming the zygote. It is well documented that fertilization triggers Ca^2+^ waves that lead to the prevention of polyspermy, oocyte activation, and meiotic resumption. Specifically, the oocyte resumes and completes meiosis II: half of the sister chromatids are segregated into the small second polar body, while the remaining chromatids that are retained in the large ovum decondense and form the female pronucleus. The chromosomes contained in the sperm head decondense and form the male pronucleus.

After DNA replication, the maternal and paternal genomes reconstitute the diploid status in a process called syngamy, chromosomes condense, and the first mitotic division of the new individual commences. Firstly, the zygote develops into the 2-cell embryo. Thereafter, the embryonic cells (the blastomeres) form a small cluster of cells referred to as the morula, which soon undergoes a process called “compaction”, forming a more uniform surface of the embryo. The outer cells develop into the trophectoderm. Subsequently, during the process of blastulation, a fluid-filled cavity, the blastocyst cavity, develops inside the trophectoderm, and the inner cells, forming the inner cell mass (ICM), gather at one pole of the embryo, which is now known as a blastocyst. The trophectoderm will participate in placenta formation while the ICM gives rise to the embryo proper. The blastocyst expands, hatches from the zona pellucida, and later, towards the end of blastulation, the ICM forms an internal and external cell layer—the hypoblast and epiblast, respectively—to establish the embryonic disc. Implantation and placental development in the uterus occur in a species-specific manner.

### 5.2. Overview of the Ca^2+^ and ROS Signaling Interplay in Animal Cells

In animals, as in plants, a mutual interplay between Ca^2+^ and ROS signaling has emerged in the last decades: Ca^2+^ signaling is essential for ROS production and, on the other hand, ROS can affect cellular Ca^2+^ signaling. Likewise, it is widely accepted that physiological (i.e., subtoxic) levels of ROS act as signaling molecules by oxidizing proteins, lipids, and nucleotides [233]. One of the main effects of ROS is the partially reversible oxidation of cysteines, leading to the formation of disulfide bonds, as well as further oxidation products, such as sulfenic, sulfinic, and sulfonic acids, which in turn modulate protein conformation and activity. However, a further increase in ROS concentration affects cellular homeostasis, leading to oxidative stress response and cellular damage. The specific roles of Ca^2+^ and ROS, as well as their interplay in physiological and pathological conditions, have been extensively reviewed (for example, see [234,235,236,237,238,239,240]). Here, we present a brief overview of the relevant concepts in animal cells, in order to introduce the current view on Ca^2+^/ROS interplay during early embryonic development in mammals.

Ca^2+^ ions can allosterically regulate the activity of many proteins and enzymes. Therefore, as already discussed for plants, Ca^2+^ ions are implicated in a high variety of functions in animal cells. Ca^2+^ signaling pathways are key in many crucial biological processes, such as cell survival, proliferation and death, contraction, secretion, metabolism, and regulation of gene expression. Animal cells have evolved complex mechanisms to control the fluctuations of cytosolic Ca^2+^ ions and, in turn, Ca^2+^-elicited cellular functions. The concentration of cytosolic Ca^2+^ can be altered by the controlled mobilization from two Ca^2+^ sources, the extracellular microenvironment, and the intracellular stores, namely, the endoplasmic reticulum (ER)—which is the main Ca^2+^ store—the Golgi apparatus, and the mitochondria. Animal cells exert this function through the coordinated activity of pumps and channels as well as Ca^2+^-binding signaling molecules, enzymes, and buffering proteins. One of the most common pathways for mammalian cells to trigger Ca^2+^ signaling is initiated by ligand-dependent G protein-coupled receptors, causing the synthesis of 1,4,5-inositol trisphosphate (IP3) and IP3-dependent opening of Ca^2+^ channels of the IP3 receptor (IP3R) family at the ER membrane (for a recent review, see [241,242,243]). Typically, resting animal cells have a low concentration of cytosolic Ca^2+^ (around 100 nM), while a rise (up to 2–3 µM) in its concentration stimulates specific cellular function (reviewed in [239,243]). Once Ca^2+^ ions have carried out their signaling functions, they are rapidly removed from the cytosol by extrusion to the extracellular space or by intracellular compartmentalization [243]. Interestingly, a comparative analysis of Ca^2+^ ion transport proteins in plants and animals revealed that the Ca^2+^ elements with basic functions in cell responses (CNGC, iGlu receptor, Ca^2+^ATPase, and Ca^2+^/Na^+^-K^+^ ion exchanger) are basically conserved between plants and animals, while the genes specific for muscle and nerve Ca^2+^ signal transduction systems (VDCC, IP3 receptor, ryanodine receptor) are very different [244]. 

Many ROS-generating systems in a cell can be modulated by Ca^2+^, including those active in mitochondria and those that operate extra-mitochondrially, such as NADPH oxidases, uncoupled nitric oxide synthase, cytochrome P450s, cyclooxygenases, and many others [245]. 

For the purpose of this review, the role of mitochondria in the control of Ca^2+^ homeostasis is particularly relevant [234,236,238,246,247] as mitochondria are an important source of ROS in the gonads and their functionality is pivotal in gametes and embryos [248,249]. After the discovery that mitochondria accumulate Ca^2+^ [250,251], a series of studies revealed that the intra-mitochondrial Ca^2+^ concentration remains low in resting animal cells, but it rapidly increases upon cell stimulation, and that this is possibly due to the close proximity of mitochondria with Ca^2+^ channels in the ER that elicits the rise in cytosolic Ca^2+^ [252,253,254,255] (reviewed in [239]). The mitochondrial Ca^2+^ uniporter (MCU) and the voltage dependent anion channels (VDACs) are primarily responsible for the function that this organelle exerts in Ca^2+^ homeostasis (reviewed in [239]).

In mitochondria, Ca^2+^ uptake is in turn involved in controlling the rate of ATP production by stimulating enzymes of the Krebs cycle and oxidative phosphorylation. Nevertheless, Ca^2+^ control of ROS production is very complex, and the interactions depend on the cell and tissue types. Since mitochondrial ROS are produced at different sites of the electron transport chain (mostly complex I and III), it is logical to think that high metabolic rates are associated with high ROS production [256]. Nevertheless, it must be highlighted that, within a certain level of Ca^2+^, the metabolic state (i.e., membrane potential) of the mitochondria itself seems to determine the effects of Ca^2+^ uptake on the mitochondrial ROS level. However, in other circumstances, when mitochondria are overloaded with Ca^2+^, ROS production is no longer dependent upon the mitochondrial metabolic state. Furthermore, in the presence of other factors (such as oxidative stress, high phosphate concentrations, and low adenine nucleotide concentration), excessive accumulation of mitochondrial Ca^2+^ has negative consequences: ATP production is impaired, and the production and sustained opening of the high mitochondrial permeability transition pore (mPTP) is induced [257]; this, in turn, induces a dramatic increase in mitochondrial membrane permeability, its depolarization, and ultimately mitochondrial swelling. As a consequence, a further increase in mROS production, the release of cytochrome c, and, ultimately, apoptosis occur [239,257] (reviewed in [239]).

In conclusion, emission of ROS from mitochondria is the net result of ROS production at the electron transport chain (ETC) and their elimination by antioxidative enzymes [246] and the mutual interplay between Ca^2+^ and ROS, which is quite complex in animal cells. This is also reflected in the relevance of Ca^2+^/ROS crosstalk in the physiopathology of the heart, skeletal muscle, neurons, and, eventually, in aging and cancer (reviewed in [239,258]).

### 5.3. ROS/Ca^2+^ Signaling in Mammalian Early Embryonic Development

#### 5.3.1. Ca^2+^ Signaling

Ca^2+^ exerts a pivotal role in animal gametogenesis, fertilization, and embryonic development (for a recent review, see [259]). Initiation of the development is triggered by a Ca^2+^-activating signal, which has been broadly studied in both mammalian and non-mammalian species [260,261,262]. All of the knowledge in mammals derives from studies in mice. However, pioneering studies in animal models, such as in fish and sea urchin, have been fundamental to our current knowledge on the role of Ca^2+^ waves in mammalian development (reviewed in [260]). 

As anticipated, in mammals, the oocyte acquires the capability to be fertilized during oocyte growth and subsequent maturation, when the immature prophase I-arrested oocyte resumes meiosis, completes the first meiotic division by extruding the first polar body, and arrests again at the metaphase stage of the second meiotic division (MII), when fertilization will eventually occur (see schematic Figure 3B for a developmental schematic overview of embryogenesis in mammals). Although the immature oocyte is capable of generating Ca^2+^ waves, which persist for a few hours and cease when the nuclear envelope breaks down after meiotic resumption [259,263,264,265], the precise biological significance of this phenomenon is still unclear (albeit it could relate to mitochondrial activity, see below). Interestingly, several studies in rat oocytes indicate that a moderate increase in Ca^2+^ and ROS, together with a transient decrease in cyclic AMP content, destabilize MPF, thus promoting meiotic resumption of the prophase I-arrested oocyte [266,267,268], which occur spontaneously (i.e., independently from the hormonal stimuli) when the oocytes are isolated from ovarian follicles. Moreover, during maturation, the cytoplasm of the oocyte is remodeled. Experimental evidence suggest that the precise machinery required to develop the characteristic Ca^2+^-dependent series of events that occur at fertilization (see below) is acquired during maturation [264]. These changes include the reorganization of the ER—the major Ca^2+^ store in the oocyte—the increase in intracellular Ca^2+^ stores, the changes in IP3R 1 expression pattern, as well as redistribution of the Ca^2+^-binding proteins [259,264,265,269,270,271]. 

At fertilization, when the sperm and the MII stage-arrested oocyte (egg) fuse, the sperm delivers the diffusible phospholipase C ζ (PLC ζ) into the egg cytosol. PLC ζ then hydrolyzes phosphatidylinositol 4,5-bisphosphate (PIP2) into IP3, which binds its receptor, IP3R1, in the ER, which, in turn, triggers Ca^2+^ release into the cytosol [272,273]. The Ca^2+^ concentration then oscillates periodically, generating Ca^2+^ waves, which last for several hours after sperm entry [262]. Activation of several pathways and enzymes by Ca^2+^ oscillation leads to several fundamental events in the fertilized egg, which are collectively referred as to oocyte activation, such as meiotic resumption (i.e., exit from the MII arrest), completion of meiosis II, formation of the pronuclei, and exocytosis of the cortical granules to prevent polyspermy. As in other mammalian cells, the Ca^2+^ concentration returns to its baseline by extrusion through the plasma membrane and/or re-loading in the ER, through the activity of the Ca^2+^ ATPases (PMCAs) and/or Na^+^/Ca^2+^ exchanger (NCX) at the plasma membrane, and the sarco-ER Ca^2+^ATPases (SERCAs) in the ER, respectively (see Figure 1 of [265]). Mitochondria and the Golgi apparatus also contribute through the activity of the mitochondrial Ca^2+^ uniporter (MCU) and the secretory-pathway Ca^2+^-transport ATPases (SPCAs), which take up Ca^2+^ into these organelles. Nevertheless, a role as a Ca^2+^ store does not seem to be the main function of mitochondria in fertilized eggs, but rather mitochondrial Ca^2+^ uptake seems more related to energy production [274,275]. The precise molecular mechanisms that drive all the processes described above are starting to be elucidated and are the subject of intense investigation (reviewed in [259,265]). 

Clearly, the control of Ca^2+^ waves in fertilized oocytes has wide implications in mammalian fertility. Very recently, CRISPR/Cas9 technology was used to generate PLCζ KO mice. These studies provided the definitive evidence that PLCζ is the physiological trigger of the Ca^2+^ oscillations in mammals [276,277]. In addition, studies in humans identified a mutation in the gene encoding for PLCζ, which led to a reduced ability to generate Ca^2+^ oscillations in fertilized eggs [278,279,280]. 

#### 5.3.2. ROS Signaling

For many years, ROS have been considered almost exclusively as detrimental compounds in the field of mammalian reproductive biology. Intense research has been conducted on this subject, and nowadays almost 10,000 publications are found on PubMed when the database is mined for the keywords “oxidative stress and mammalian reproduction”. Still today, even though physiological roles of ROS are widely recognized [249], their detrimental effect is a very hot topic among reproductive scientists [281,282]. This is mainly because oxidative stress, caused by elevated ROS production and/or a decreased antioxidative system, is considered one of the predominant causes of both male and female infertility. Moreover, oxidative stress profoundly impacts the efficiency of assisted reproductive technologies (ART), which are widely used for the treatment of human infertility, in animal breeding, as well as for the preservation of endangered species. ROS production is stimulated during all the steps of in vitro embryo production, such as in vitro oocyte maturation, fertilization, and early embryo culture. Moreover, gametes and embryo freezing are largely used in ART, where ROS generation is particularly challenging. As a consequence, a plethora of studies have focused on the assessment of the effects of antioxidant compounds on in vitro embryo development, and to find the most suitable culture conditions to recapitulate fertilization and early embryonic development in vitro. Just as an example, early embryos are usually cultured under low oxygen tension (5%) to minimize oxidative stress. However, excess antioxidants were found to be not only ineffective but sometimes deleterious. This may be attributed to a disturbance in the signaling functions of ROS by the excess of antioxidants [249]. 

Nevertheless, the physiological milieu in which the female gametes are generated and then fertilized is, per se, rich in ROS. As a matter of fact, the ovary is an endocrine organ that generates ROS during steroidogenesis. Ovulation, the process by which the mature egg is released in the oviduct for fertilization, is recognized as an inflammatory-like reaction and many proinflammatory agents, such as interleukins and tumor necrosis factor, have physiological roles in the ovarian follicle [283]. Thus, antioxidant processes are set in place in the ovary to protect gametes from oxidative compounds. Many studies have demonstrated that when the function of the antioxidant machinery is diminished, such as in ageing or when antioxidative enzymes are depleted experimentally, fertility is impaired [284]. Furthermore, studies in mice have shown that, in the oocyte, ROS are produced by mitochondria and by oocyte oxygenase [285]. Notably, defective mitochondrial functions are generally associated with poor oocyte developmental competence and meiotic errors. Thus, much is known on the “bad side of ROS” in the ovary and in female egg development. On the contrary, ROS-mediated signaling events in oocytes are less known, one of the limitations being the high amount of ROS that are administered/generated in vitro and the difficulties to study and visualize this phenomenon in living organisms.

In males, spermatogenesis is also prone to the damaging effects of ROS. A sophisticated array of antioxidant systems that include both enzymes and free radical scavengers is set in place in testis to limit the detrimental effects of ROS [249,286,287]. However, the physiological functions for ROS have been reported also in the testis (reviewed in [288]).

ROS are essential signals involved in the regulation of spermatogenesis, steroidogenesis, and sperm function. Specifically, in the sperm, they trigger morphological changes required for sperm maturation and modulate crucial processes involved in the attainment of sperm fertilizing ability, such as capacitation, hyperactivation, acrosome reaction, and sperm–oocyte fusion (reviewed in [288]). For example, physiological levels of ROS are crucial for sperm maturation that occur in the epididymis, the anatomical site where spermatozoa acquire motility, and where chromatin condensation and plasma membrane remodeling occur. During spermiogenesis, histones in chromatin are replaced by protamines that are rich in Cys residues; in the epididymis, H_2_O_2_ and antioxidants, such as glutathione peroxidase 4 and 5 (GPX4, GPX5) and peroxiredoxin 6 (PRDX6), contribute to the formation of sulfhydryl bridges between the cysteine residues of the protamines, resulting in chromatin condensation in the sperm head (reviewed in [288]).

Recently, the knowledge on the role of H_2_O_2_ in metazoan development has been revisited in the frame of the morphogenetic process during embryogenesis, regeneration, and stem cell differentiation [7]. What emerges is that “redox signaling interacts directly or indirectly with most of the signaling pathways that control embryonic development”. However, as the authors conclude, “We are only starting to perceive the tip of the iceberg”. Similarly, ROS signaling during early mammalian embryogenesis (i.e., before implantation) is far from being deciphered. As already mentioned, oxidative stress is detrimental for early embryos. It has been shown that oxidative stress associated with in vitro culture induces developmental arrest (i.e., block of the cell cycle) and cell death. Studies in *SOD* KO mice suggest that, under oxidative stress conditions, ROS act by regulating the expression of genes of the cell cycle machinery [249]. Nevertheless, studies in mouse and bovine early embryos have shown increased concentration of ROS at critical stages of development, and the assessment of embryo metabolism confirmed that ROS are key at fertilization and early embryo development [285,289,290,291]. In bovine, in particular, the assessment of oxygen consumption and ROS production at the time of fertilization and cell cleavage have suggested that both processes have a regulatory role. Importantly, these studies, together with the ones that have established a correlation between oxygen consumption and Ca^2+^ waves [274,275,290,292], led to the suggestion that mitochondrial activity is stimulated by Ca^2+^ oscillation at fertilization in mammals [291]. 

#### 5.3.3. Ca^2+^/ROS Signaling Interplay in Gametes and Early Embryos

Very little is known about the Ca/ROS interplay during gametogenesis and early development, especially mechanistically. However, two very recent studies clearly indicate that the available technologies might be finally able to answer many unresolved questions, and likely accelerate the discoveries of new key processes in development.

The first study, conducted in a mouse model, demonstrated that constitutive IP3R1-mediated Ca^2+^ release reduces Ca^2+^ store content and stimulates mitochondrial metabolism in prophase I immature oocytes [259]. As already anticipated, the immature oocyte is capable of generating Ca^2+^ waves, which persist for a few hours and cease when the nuclear envelope breaks down after meiotic resumption [259,263,264,275]. By using technology, such as the microinjection of fluorescent probes targeted at mitochondria and advanced imaging systems to detect Ca^2+^ oscillation using the Fura-2 probe, Wakai and Fissore found that Ca^2+^ stored in the ER of immature oocytes constitutively “leaks” through the IP3R1 [265]. The Ca^2+^ “leak” ceases around the time when the oocyte resumes meiosis and the nuclear envelope breaks down, which is when the accumulation of Ca^2+^ in the cellular stores, in preparation for fertilization, is firstly seen. Strikingly, during Ca^2+^ oscillation, Ca^2+^ is transferred to the mitochondria, where it stimulates metabolisms and increases the levels of ATP [259]. Clearly, this study opens a new area of investigation on the possible role of the Ca^2+^/ROS interplay in female gametogenesis.

The second study took advantage of a transgenic *Xenopus laevis* that ubiquitously expressed the H_2_O_2_ sensor HyPer, including the eggs of transgenic females [293]. Using this model, the authors demonstrated that, at fertilization, the Ca^2+^ waves induced by the sperm–egg fusion triggers a rapid increase in ROS levels, which in turn oscillate with each cell division. Furthermore, using inhibitors of complexes II, III, or IV of the electron transport chain, they also demonstrate that these enzymes are responsible for ROS generation. Importantly, their data also suggest that Ca^2+^-induced ROS signaling might control the onset of cell division, as inhibition of mitochondrial ROS production in early embryos results in cell cycle arrest, in part, via ROS-dependent regulation of Cdc25C activity. This is important in that cdc25 activates the cyclin B/cyclin-dependent kinase complex that plays a key role in cell cycle regulation to induce entry into mitosis [293]. Since the Ca^2+^/ROS interplay has been suggested also in mammalian fertilization (see paragraph 4.3.2) and early embryos, this study sets the stage for a new area of research in mammalian early development.

## 6. Conclusions and Future Remarks 

In this review, we have revisited the literature concerning ROS signal transduction in sexual reproduction in plants and animals. Although these two systems are, apparently, biologically far different, common strategies concerning sexual reproductive patterns are shared among them. Probably this is due to the fact that sexual reproduction evolved in eukaryotes well before the divergence of plants and animals [294]. As a matter of fact, transcriptome analysis performed of plant female gametophyte gene expression revealed common molecular pathways affecting gamete (syngamy) and nuclear fusion (karyogamy) between those lineages [295]. Several reproductive patterns of angiosperms evolved similar to those in mammals, for instance, the embryo development surrounded by a maternal environment providing nutrients, the programmed arrest of the mature gamete before fertilization event, the presence of common parental imprinting evolved in both groups, and a selection based on male–male competition [35,296,297,298,299,300,301,302]. In animals, ROS is involved in sperm activation and in egg activation and fertilization [214,284,303,304]. In plants, ROS burst is necessary for proper pollen development, pollen tube rupture, and sperm release [174,222,305]. A role for Ca^2+^ in sperm production and maturation as well as for female–male gamete interaction has been well documented both in animal and plant systems [261,306,307,308,309,310]. As described in this review, new analytical techniques are being developed during the last years—and are still evolving—such as the use of ratiometric genetically encoded sensors for the measurement of Ca^2+^, H_2_O_2_ and sensors for testing the redox status of GSH [86,119,120]. As discussed, most are already available for animal cells, which allow the in vivo measurements of these parameters in them. In plants, the role of most of the players affecting ROS homeostasis in sexual reproduction and embryo development has yet to be elucidated. As in mammals, single-cell and other tissue specific transcriptome data are now available for specific reproductive structures of plants, such as the female gametophyte and the embryo [217,311,312,313,314,315]. Comparative studies in plant and animal developmental processes, exploring the vast mass of information, would help to build comparative mechanistic models that take into account the similarities shared by both lineages, which will elucidate the novel key players affecting ROS and Ca^2+^ signaling. Further advances and comparative studies on the role of ROS/Ca^2+^ should provide new clues into the communication mechanisms between zygotic and maternal tissues in plants and animals, which are relatively poorly described in both lineages.

In agriculture, the elucidation of the complex signaling pathways triggered by plant exposure to various environmental stresses and involving ROS and Ca^2+^ could support the identification of strategies to improve plant resilience to both biotic and abiotic stresses. Climate changes and global warming are posing new unprecedented challenges to crop production; efforts to understand plant resilience to environmental stresses are currently being made by different research groups [158,316]. As an example, the priming effects of high CO_2_ on plant defenses through redox signaling pathways have been recently uncovered [317]. Advancements in the understanding of the actions and signaling of ROS in developing and germinating seeds, and their aging process [318,319], also might support existing protocols applied in the various seed banks and enhance success rates in seed preservation and germination.

## Figures and Tables

**Table 1:** The genes affecting ROS and Ca^2+^ signaling cited in this work.

Gene ID	Full Gene Name	Plant Structure Affected	Reference	Impact on ROS and Development
***At2g47270***	*UPBEAT1* (*UPB1*), a transcription factor with a bHLH domain	Root differentiation	[164,165]	Regulates the expression of a set of peroxidases that modulate the balance of ROS between the zones of cell proliferation and the zone of cell elongation where differentiation begins. Disruption of UPB1 activity alters this ROS balance, leading to a delay in the onset of differentiation.
***At5g53540***	*P-loop NTPase APP1*	Root differentiation	[166,167]	Encodes a P-loop NTPase APP1. The disruption of APP1 is accompanied by a reduction in ROS level, a rise in the rate of cell division in the quiescent center (QC) and the promotion of root distal stem cell (DSC) differentiation.
***At4g11690***	*ABA OVERLY SENSITIVE MUTANT*(*ABO8*), a pentatricopeptide repeat (PPR) protein	Root differentiation	[168,169]	Abo8 mutants accumulate more ROS in root tips than the wild type.
***At5g40770***	*PROHIBITIN* 3 (*PHB3*)	Root differentiation	[168,170,171]	PHB3 coordinates cell division and differentiation in the root apical meristem via ROS-dependent signaling.
***At2g12646***	*RGF1-INDUCIBLE TRANSCRIPTION FACTOR 1* (*RITF1*)	Root meristem	[172,173]	Controls root meristem size through ROS signaling.
***At3g10920***	*MANGANESE SUPEROXIDE DISMUTASE* (*MSD1*)	Pollen	[174,175]	Female gametophytic mutant impaired in mitochondrial manganese-superoxide dismutase (MSD1) displays high levels of ROS detectable in the central cell and micropylar cells.
***At5g63290***	*ATHEMN1*, *HEMN1*	Female gametophyte	[175,176]	*athemn1* mutant defective in tetrapyrrole biosynthesis which had increased ROS accumulation in developing embryo sacs and defects in female gametophyte development with embryo sacs displaying unfused polar nuclei.
***At5g43285***	*LURE1.1*	Synergid cell at female gametophyte	[177,178,179]	Encodes a cysteine-rich peptide that acts as a pollen tube attractant guiding pollen tubes to the ovular micropyle.
***At4g08869***	*LURE1.7*	Pollen tube	[177,178,179]	Encodes a defensin-like family protein. Pollen tube emergence accelerator that favors conspecific pollen over pollen from other species and thus promotes reproductive isolation.
***At4g08875***	*LURE1.8*	Pollen tube	[177,178,179]	Encodes a defensin-like family protein. Pollen tube emergence accelerator that favors conspecific pollen over pollen from other species and thus promotes reproductive isolation.
***At3g51550***	*FER*, *FERONIA*	Synergid cell at female gametophyte	[180,181,182]	Receptor-like kinase involved in pollen tube reception.
***At3g46290***	*HERCULES RECEPTOR KINASE 1* (*HERK1*)	Pollen tube	[180,183]	Receptor-like kinase involved in pollen tube reception.
***At3g04690***	*ANXUR1* (*ANX1*)	Pollen tube growth	[184,185]	Receptor-like kinase involved in pollen tube reception.
***At2g17430***	*NORTIA* (*NTA*, *MLO7*)	Pollen tube reception	[182]	Studies performed with the *Arabidopsis* synergid-expressed gene NORTIA have shown how calcium oscillation in synergids is fundamental to proper pollen tube reception. *nta* mutants affected in Ca^2+^ display the pollen tube overgrowth phenotype.
***At2g33670***	MILDEW RESISTANCE LOCUS O 5 (MLO5)	Stigma, anther, and pollen grains	[186]	MLO5 and MLO9 selectively recruit Ca^2+^ channel CNGC18-containing vesicles to the plasma membrane through the R-SNARE proteins in order to modify Ca^2+^ gradients in the pollen tube.
***At1g42560***	MILDEW RESISTANCE LOCUS O 9 (MLO9)	Pollen	[186,187,188]	MLO5 and MLO9 selectively recruit Ca^2+^ channel CNGC18-containing vesicles to the plasma membrane through the R-SNARE proteins in order to modify Ca^2+^ gradients in the pollen tube.
***At2g44110***	MILDEW RESISTANCE LOCUS O 15 (MLO15)	Seedlings, root tips, and flower structure	[186,187,188]	MLOs; together with MLO5 and MLO9, MLO15 is required for proper pollen tube sensitivity to ovule signals in *Arabidopsis thaliana*.
***At5g60010***	RESPIRATORY BURST OXIDASE HOMOLOG H (RBOHH)	Pollen	[189]	ROS production by RbohH and RbohJ is essential for proper pollen tube tip growth. Double mutant pollen tubes cease their growth and burst in vitro and fail to reach the female gametophytes in vivo.
***At3g45810***	RESPIRATORY BURST OXIDASE HOMOLOG J (RBOHJ)	Pollen	[189]	ROS production by RbohH and RbohJ is essential for proper pollen tube tip growth. Double mutant pollen tubes stop growth, burst in vitro and fail to reach the female gametophytes in vivo.
***AT1G63990***	*SPORULATION 11 (SPO11)*	*Floral organs*	[35,190]	*SPO11 is a DNA topoisomerase whose activity is exposed to redox regulation. SPO11 is required for meiotic recombination. Plants homozygous for atspo11-2 exhibit a strong sterility phenotype and this is associated with severe defects in synapsis during the first meiotic division and reduced meiotic recombination.*

**Table 2:** Expression of the genes involved in ROS metabolism in Arabidopsis female gametophyte cells; the data for selected genes were extracted from Table S1 of reference [217] and presented as Supplementary Table S1. The average gene expression value was calculated for each cell type. Red, high expression level; yellow, medium expression level; white, no expression detected; on a relative scale.

Gene ID	Short_Description	Egg Cell	Central Cell	Synergid Cell
*At1g20630*	Catalase 1	52.04	**4757.34**	**265.66**
*At4g35090*	Catalase 2	0.00	495.80	0.00
*At1g20620*	Catalase 3	9.72	177.36	0.00
*At1g08830*	Copper/Zinc Superoxide Dismutase 1	6.62	911.31	25.49
*At1g09090*	Respiratory Burst Oxidase Homolog B	0.00	2.85	0.00
*At1g12520*	Copper Chaperone For SOD1	5.02	6.81	0.00
*At1g14920*	GAI /GRAS Family Transcription Factor Family Protein	3.14	1.10	0.00
*At1g32230*	WWE Protein-Protein Interaction Domain Protein Family	20.53	258.70	3.41
*At1g66350*	RGA-Like 1	0.00	1.01	0.00
*At1g17020*	Senescence-Related Gene 1	19.50	3.59	0.00
*At4g12420*	Cupredoxin Superfamily Protein	10.86	7.81	0.00
*At4g39830*	Plant L-Ascorbate Oxidase	0.00	16.79	0.00
*At5g21100*	Plant L-Ascorbate Oxidase	0.00	2.26	0.00
*At5g21105*	Plant L-Ascorbate Oxidase	3.20	7.96	8.67
*At2g25080*	Glutathione Peroxidase 1	13.18	272.94	0.00
*At2g31570*	Glutathione Peroxidase 2	1.82	**5543.76**	0.00
*At2g43350*	Glutathione Peroxidase 3	10.49	358.55	102.44
*At2g48150*	Glutathione Peroxidase 4	0.00	25.73	0.00
*At3g63080*	Glutathione Peroxidase 5	5.18	83.62	66.53
*At4g11600*	Glutathione Peroxidase 6	**153.48**	**4766.73**	**206.43**
*At4g31870*	Glutathione Peroxidase 7	0.00	12.64	0.00
*At1g63460*	Glutathione Peroxidase 8	13.60	259.42	12.65
*At2g30860*	Glutathione S-Transferase PHI 9	0.00	0.00	0.00
*At5g27380*	Glutathione Synthetase 2 GSH2	1.82	402.09	0.00
*At4g23100*	Glutamate-Cysteine Ligase GSH1	65.25	1411.02	0.00
*At4g33670*	Gal-DH NAD(P)-Linked Oxidoreductase Superfamily Protein	0.00	273.91	0.00
*At3g55590*	Glucose-1-Phosphate Adenylyltransferase Family Protein	3.42	131.82	53.29
*At2g39770*	GDP-Mannose VTC1	2.18	326.29	0.00
*At3g02870*	Inositol Monophosphatase Family Protein VTC4	**124.00**	616.27	0.00
*At5g55120*	Galactose-1-Phosphate Guanylyltransferase VTC5	2.88	139.24	16.42
*At4g26850*	Mannose-1-Phosphate Guanylyltransferase VTC2	6.67	118.31	0.00
*At3g47930*	GalDH L-galactono-1,4-lactone dehydrogenase	0.00	204.95	5.26
*At5g56490*	D-arabinono-1,4-lactone oxidase GulLO	1.26	182.31	3.41
*At5g28840*	GDP-D-mannose 3’,5’-epimerase GME	86.90	395.34	0.00
*At1g32300*	D-arabinono-1,4-lactone oxidase family protein	0.00	0.00	0.00
*At4g39120*	IMPL2 myo-inositol monophosphatase like 2	4.97	163.22	0.00
*At1g31190*	IMPL1 myo-inositol monophosphatase like 1	2.17	212.95	0.00
*At2g46740*	D-arabinono-1,4-lactone oxidase family protein	0.00	13.04	0.00
*At2g46750*	D-arabinono-1,4-lactone oxidase family protein	0.00	0.00	16.42
*At2g46760*	D-arabinono-1,4-lactone oxidase family protein	0.00	0.00	0.00
*At5g11540*	D-arabinono-1,4-lactone oxidase family protein	0.00	784.19	0.00
*At5g56470*	FAD-dependent oxidoreductase family protein	2.93	4.91	0.00
*At5g56490*	D-arabinono-1,4-lactone oxidase family protein	1.26	182.31	3.41
*At4g32770*	Tocopherol cyclase / vitamin E deficient 1 (VTE1)	0.00	175.02	0.00
*At3g63410*	S-adenosyl-L-methionine-dependent methyltransferase	0.00	414.27	0.00
*At1g64970*	Gamma-tocopherol methyltransferase	0.00	0.00	0.00
*At2g18950*	Homogentisate phytyltransferase 1	0.00	0.00	0.00
*At3g11945*	Homogentisate prenyltransferase	0.00	180.24	5.89
*At1g06570*	Phytoene desaturation 1	8.55	50.43	5.26

## Data Availability

Data sharing not applicable.

## Supplementary Materials

The following are available online at https://www.mdpi.com/article/10.3390/genes12040525/s1, Table S1: Full datasets supplementary to the data which are presented in Table 2. Original data from [217].
